# Relationship between population density and viral infection: A role for personality?

**DOI:** 10.1002/ece3.5541

**Published:** 2019-08-18

**Authors:** Bram Vanden Broecke, Joachim Mariën, Christopher Andrew Sabuni, Ladslaus Mnyone, Apia W. Massawe, Erik Matthysen, Herwig Leirs

**Affiliations:** ^1^ Evolutionary Ecology Group Department of Biology University of Antwerp Antwerp Belgium; ^2^ Pest Management Center Sokoine University of Agriculture Morogoro Tanzania

**Keywords:** animal personality, arenavirus, density, density dependence, disease ecology, exploration, *Mastomys natalensis*, Morogoro virus, multimammate mice, stress sensitivity

## Abstract

Conspecific density and animal personality (consistent among‐individual differences in behavior) may both play an important role in disease ecology. Nevertheless, both factors have rarely been studied together but may provide insightful information in understanding pathogen transmission dynamics. In this study, we investigated how both personality and density affect viral infections both direct and indirectly, using the multimammate mice (*Mastomys natalensis*) and Morogoro arenavirus (MORV) as a model system. Using a replicated semi‐natural experiment, we found a positive correlation between MORV antibody presence and density, suggesting that MORV infection is density‐dependent. Surprisingly, slower explorers were more likely to have antibodies against MORV compared to highly explorative individuals. However, exploration was positively correlated with density which may suggest a negative, indirect effect of density on MORV infection. We have shown here that in order to better understand disease ecology, both personality and density should be taken into account.

## Graphical abstract

Most studies concerning personality and its effect on parasitic or viral infection probability found that highly explorative individuals were more likely to acquire infection. We have found the opposite pattern in *Mastomys natalensis*, where individuals with antibodies against the Morogoro virus (MORVab) were significantly less explorative than individuals without antibodies against this virus.

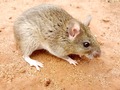

## 1 INTRODUCTION

In the last couple of decades, there is an increase of emerging infectious diseases worldwide of which 60% are zoonotic and originating from wildlife (Daszak, 2000; Jones et al., 2008; Karesh et al., 2012). To understand these diseases, a more profound knowledge of the transmission mechanisms within wild populations is needed. One of the prominent factors that could affect transmission is host density, especially for directly transmitted pathogens (Altizer et al., 2006; Anderson & May, 1978; Davis et al., 2004; Davis & Calvet, 2005). This density‐dependent infection probability, however, differs among individuals resulting in infection heterogeneity among individuals (VanderWaal & Ezenwa, 2016; Woolhouse et al., 1997) and could partially be attributed to consistent behavioral differences between individuals across time and/or contexts, currently referred to as animal personality (Carere & Maestripieri, 2013; Réale, Reader, Sol, McDougall, & Dingemanse, 2007). Indeed, there is a growing body of evidence that personality affects infection probability, as several studies found a positive correlation among exploration, boldness, activity, and parasite/pathogen load (Barber & Dingemanse, 2010; Bohn et al., 2017; Boyer, Réale, Marmet, Pisanu, & Chapuis, 2010; Dizney & Dearing, 2013; Patterson & Schulte‐Hostedde, 2011). For example, bolder deer mice (*Peromyscus maniculatus*) are three times more likely to be infected with Sin Nombre virus and responsible for most of the transmission events (Clay, Lehmer, Previtali, St Jeor, & Dearing, 2009; Dizney & Dearing, 2013).

**Figure 1:**
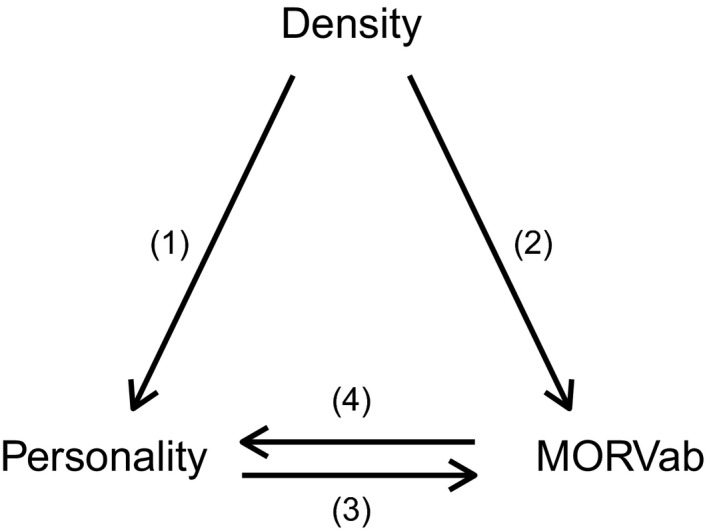
Schematic view of the potential interactions among density, personality, and the amount of individuals with antibodies against the Morogoro virus (MORVab). The numbers refer to different predictions discussed in the text

In order to understand pathogen transmission, both density and personality should therefore be incorporated into one framework (Figure 1). This may allow us to disentangle direct effects of density on infection probability as well as indirect effects due to a relationship between density and personality. Indeed, population density and density‐dependent processes are important in the evolutionary framework of animal personality since they may lead to fluctuating selection, which is considered to be one of the major evolutionary mechanisms responsible for the existence and maintenance of personality (Dingemanse & Wolf, 2010; Wolf, Doorn, Leimar, & Weissing, 2007). For example, the survival probability of slow exploring great tits (*Parus major*) increases with density while that of fast explorers decreases (Nicolaus, Tinbergen, Ubels, Both, & Dingemanse, 2016). Similar results have been found in the common lizard (*Zootoca vivipara*) where at low density, survival is higher in more social and less active individuals compared to less social and more active individuals (Le Galliard, Paquet, & Mugabo, 2015).

Density‐dependent processes could affect personality at both the between‐ and within‐individual level (Dingemanse & Dochtermann, 2013). A correlation at the between‐individual level implies that the composition of the population with respect to personality types changes when density changes. For instance, aggressiveness in both the prairie vole (*Microtus ochrogaster*) and the meadow vole (*M. pennsylvaticus*) differs significantly between populations at different phases in the population cycle (Krebs, 1970). Similar results have been found in bank voles (*Myodes glareolus*; Korpela, Sundell, & Ylönen, 2011) and red squirrels (*Sciurus vulgaris*; Haigh, O'Riordan, & Butler, 2017). On the other hand, a correlation between personality and density at the within‐individual level would be expected in environments with strong density fluctuations, leading to changes in resource availability and the social environment (Borremans et al., 2016). Individuals might adjust their behavior in these conditions (i.e., plasticity; Dingemanse & Wolf, 2010) potentially increasing their fitness at both low and high densities (Dingemanse & Wolf, 2013). However, phenotypic plasticity is costly, both energetically and in terms of fitness and should therefore only exist if the benefits are larger than the costs (Auld, Agrawal, & Relyea, 2010; DeWitt, Sih, & Wilson, 1998; Fischer, Doorn, Dieckmann, & Taborsky, 2014). Most studies that investigated the relationship between density and personality compared populations with different densities, where it is impossible to disentangle the between‐ and within‐individual level response to changing density. One way to do this is by studying the same population in which density changes over time.

In this study, we used a replicated, semi‐natural, experimental setup to investigate the links among density, personality and infection probability (Figure 1) using the multimammate mouse (*Mastomys natalensis*) and Morogoro virus (MORV) as a model system. This allowed us to repeatedly measure individuals within the same populations over a density gradient. Our experimental setup was ideal to investigate how density and personality might affect infection probability for several reasons. First, because transmission is mainly horizontal and density‐dependent, we expect more individuals with MORV‐specific antibodies (MORVab, an indication of infection; Mariën, Borremans, Gryseels, Soropogui, et al., 2017; Mariën, Borremans, Gryseels, Vanden Broecke, et al., 2017) in the population with increasing host density (Borremans et al., 2011; Borremans et al., 2016; Borremans, Vossen, et al., 2015; Figure 1(2)).

Second, we have found evidence for the existence of consistent differences in exploration, (i.e., animal personality) in this species (Vanden Broecke et al., 2018). Nevertheless, while it has been hypothesized that personality may affect virus infection probability (Barber & Dingemanse, 2010; Kortet, Hedrick, & Vainikka, 2010), we found no behavioral differences among individuals with or without MORVab (Vanden Broecke et al., 2018; Figure 1(3&4)).

Third, our experimental setup allowed us to disentangle the relationship between density and personality at both the between‐ and within‐individual level (Figure 1(1)). On the between‐individual level, we expect to catch more explorative individuals with increasing density, due to the influx of juveniles into the population (Leirs, Verhagen, & Verheyen, 1993, 1994) as juveniles are more explorative than adults (Vanden Broecke et al., 2018). On the within‐individual level, we expect plasticity with changing density, since social interactions in *M. natalensis* have been shown to change plastically with density (Borremans et al., 2016). However, it is difficult to make predictions about the direction of plasticity due to a potential trade‐off between reproductive success and predation pressure. On the one hand, we would expect that individuals become more explorative with increasing density since this behavior may increase the probability of finding food leading to an increase in weight, which is correlated with reproductive success in *M. natalensis* (Kennis, Sluydts, Leirs, & Hooft, 2008). On the other hand, predation pressure has been shown to reduce activity in *M. natalensis* (Mohr, Vibe‐Petersen, Lau Jeppesen, Bildsoe, & Leirs, 2003) and increases with density (Leirs et al., 1997). Nevertheless, increased exploration would be expected if the benefits of reproductive success outweigh the costs of predation.

This is the first study which examines the combined effect of population density and behavioral heterogeneity on virus infection probability in one experiment. We were able to study the direct effects of both factors on virus transmission. Additionally, due to our experimental setup, we were able to investigate the relationship between density and personality at the between‐ and within‐individual level allowing us to look at an indirect effect of density on viral infection probability.

## 2 MATERIAL AND METHODS

### 2.1 Study species

*Mastomys natalensis* is the most common indigenous rodent in sub‐Saharan Africa and an agricultural pest species (Leirs, Verhagen, & Verheyen, 1994). Breeding is triggered by sprouting grasses (Leirs, 1994) leading to a strong correlation between reproduction and seasonal rainfall, which affects food availability and leads to strong seasonal and annual fluctuations from 20 to 500 individuals per hectare in East Africa in a couple of months (Leirs, Stuyck, Verhagen, & Verheyen, 1990; Sluydts, Crespin, Davis, Lima, & Leirs, 2007). Animals enter a growth stop at the end of the breeding season, when food availability decreases (Leirs et al., 1997, 1990). Most individuals participate only in the breeding season after the one in which they were born, since only a few animals live longer than 300 days and due to the low survival probability of adults after a breeding season (Leirs et al., 1990; Sluydts et al., 2007; Sluydts, Davis, Mercelis, & Leirs, 2009). Additionally, *M. natalensis* hosts several infectious agents, such as Lassa virus (Frame, Baldwin, Gocke, & Troup, 1970), plague bacteria (Ziwa, Matee, Kilonzo, & Hang'ombe, B.M., 2013), and Morogoro virus (MORV; Goüy de Bellocq et al., 2010; Günther et al., 2009). Transmission of MORV is believed to be mainly horizontal (Borremans et al., 2011; Mariën, 2019) via direct contacts (e.g., grooming, licking, and mating) or through indirect exposure to virus particles since viral RNA particles can be found in the blood and excretions of infected individuals (Borremans, Vossen, et al., 2015; Mariën, Borremans, Gryseels, Vanden Broecke, et al., 2017). Infection appears to be acute, followed by a lifelong immunity, although a small proportion seems to become chronically infected (Mariën, Borremans, Gryseels, Vanden Broecke, et al., 2017). Pathogenicity of MORV seems not severe on the short term (Mariën, Borremans, Gryseels, Soropogui, et al., 2017); however, a long‐term capture–mark–recapture study revealed that MORVab positive individuals have a slightly lower survival probability than MORVab negative individuals (Mariën et al., 2018).

### 2.2 Experimental setup

#### 2.2.1 Enclosures

**Figure 2:**
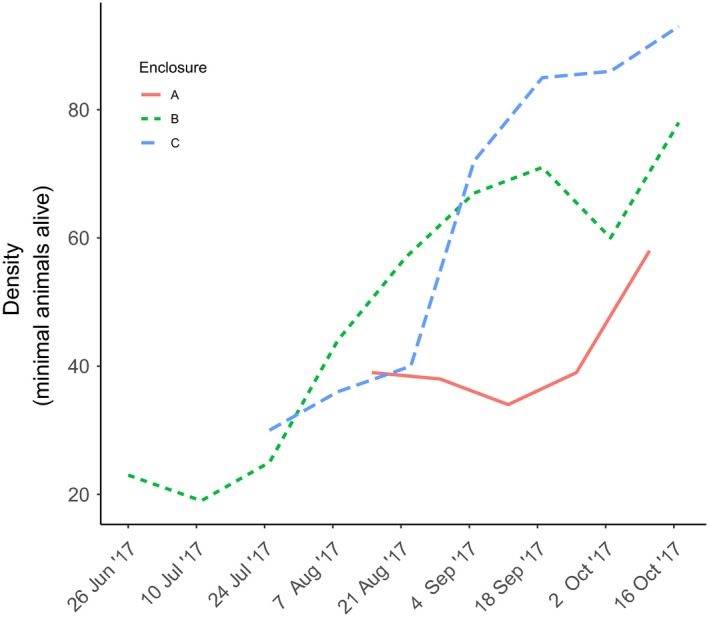
The minimal number of animals alive in each enclosure (A, B, and C), calculated for every trap session using the individual capture histories. These values were used as an estimation of density in the statistical models

The experiment was conducted in three 0.5 ha (70 × 70 m) enclosed fields (named A, B, and C) on the campus of the Sokoine University of Agriculture (SUA) in Morogoro, Tanzania from May until October 2017 (Table S4). As these enclosures were almost 10 times larger than the mean home range size of *M. natalensis* (Borremans et al., 2014), spatial behavior was unlikely to be affected by the experimental setup. The enclosures were constructed out of galvanized steel, 100 cm above and 70 cm below the ground, preventing individuals from escaping, hence increasing individual trapping success while still allowing the presence of most of the natural predators. Additionally, it allowed us to establish three different populations, serving as replicates, whose density increased over time (Figure 2). The habitat inside the enclosures consisted of a mix of grasses and shrubs similar to the rodents' natural habitat providing cover against predators and weather.

We removed all rodents from inside the enclosures before the experiment after which we randomly released rodents that had been captured in three different areas containing both fallow land and maize fields elsewhere on the campus (spaced at least 2 km from each other for spatial independence; Borremans et al., 2014). This was done to increase genetic diversity in each field and to have a precisely known starting density. Due to technical difficulties, we were not able to start with the exact same densities in each enclosures. We started the experiment after we released 39 (*N*_female adult_ = 21, *N*_female juvenile_ = 5, *N*_male adults_ = 6, *N*_male juvenile_ = 7), 23 (*N*_female adult_ = 17, *N*_male adults_ = 6), and 30 (*N*_female adult_ = 18, *N*_male adults_ = 11, *N*_male juvenile_ = 1) individuals inside enclosure A, B, and C, respectively, after which no new individuals were added (Figure 2).

During the experiment, we implemented capture–mark–recapture trapping for three consecutive nights every 2 weeks for each enclosure. We placed 100 Sherman LFA live traps (Sherman Live Trap Co.) baited with a mix of peanut butter and maize flour within each enclosure (in a 10 × 10 arrangement, with 7 m among traps) in the evening (around 16:00) and checked them in the early morning (5:00). We recorded the weight, sex, and reproductive age following Leirs et al. (1994). Individuals were uniquely marked using toe clipping (Borremans, Sluydts, Makundi, & Leirs, 2015), and blood samples were taken from the retro‐orbital sinus and preserved on prepunched filter paper (Serobuvard, LDA 22, Zoopole). These blood samples were later analyzed at the University of Antwerp for MORV‐specific IgG antibodies using immunofluorescence assay protocols described in Günther et al. (2009). The individuals were held for a maximum of 5 hr and released at the point of capture. After each trap sessions, maize kernels were thrown evenly inside each field as an additional food supply. We conducted a total of 5, 9, and 7 trapping session, respectively, for field A, B, and C and calculated the minimal animals alive during that session as an indirect measurement for density (Figure 2; Table S4). This was done using the individual capture histories where an individual was present inside the enclosure for all trap sessions between the first and last time of capture, since movements outside the enclosures was restricted.

#### 2.2.2 Behavioral recordings

Behavioral trials were conducted at the site of capture using a hole‐board test before blood sampling and toe clipping to minimize any potential effects of stress. The hole‐board test is a derivative of the open field test with holes in the floor to measure exploration independently of activity (File & Wardill, 1975; Martin & Réale, 2008b). The box (75 × 55 × 90 cm; L × W × H, respectively) was constructed out of strong white plastic with six blind holes in the bottom (Ø: 3.5 cm; depth: 6 cm) each spaced 19 cm apart from each other. The box was closed off with a lid with an infrared camera. Behavioral recordings started when the individual was inside the box, and the lid was closed and lasted for 10 min. The box was cleaned with 70% ethanol to remove scent and dirt.

All experimental procedures were approved by the University of Antwerp Ethical Committee for Animal Experimentation (LA1100135) and adhered to the EEC Council Directive 2010/63/EU and followed the Animal Ethics guidelines of the Research Policy of Sokoine University of Agriculture.

#### 2.2.3 Video analysis

Activity was measured by dividing the floor of the box into 12 squares and counting the number of times an individual changed squares during 10 min (Vanden Broecke et al., 2018). This was quantified using MTrackJ (Meijering, Dzyubachyk, & Smal, 2012), a plugin for ImageJ (Schneider, Rasband, & Eliceiri, 2012). We scored two behaviors as a measurement for exploration: the number of times the animal sniffed a hole and the number of head dips (eyes and ears disappear into one of the blind holes; File & Wardill, 1975; Martin & Réale, 2008a, 2008b), both of which were measured with JWatcher 1.0 (Blumstein & Daniel, 2007). Additionally, we measured the time spent grooming (in seconds) and number of jumps.

### 2.3 Statistical analysis

#### 2.3.1 Behavioral analysis

We conducted a total of 663 behavioral recordings on 206 unique individuals (*N*_females_ = 129, *N*_males_ = 77; *N*_enclosureA_ = 36, *N*_enclosureB_ = 91, and *N*_enclosureC_ = 79). All individuals were recorded on average three times (range = 2–8 observations) with on average 19 days between subsequent recordings (range = 12–71 days).

We used a principal component analysis (PCA) on all behaviors to reduce the number of variables and the Kaiser–Guttman criterion (eigenvalue >1; Kaiser, 1991; Peres‐Neto, Jackson, & Somers, 2005) was used to select the number of components to retain.

#### 2.3.2 Relationship between density and personality

We used linear mixed models (LMM) with a Gaussian error distribution in order to determine whether density (on both the between‐ and within‐individual level), MORVab presence as well as sex and reproductive age affected the observed variation in exploration and stress sensitivity among and within individuals. We therefore ran two LMM with the two principal components as response variables. In each model, we included reproductive age (adult or juvenile), sex (male or female), and sequence of recordings (binomial variable describing if it is the first time an individual had been recorded or subsequent recordings) to control for habituation to the test (Vanden Broecke et al., 2018). Serological data were included in the model by dividing the individuals into four groups to avoid collinearity: individuals that were (a) MORVab negative (*N* = 93) or (b) MORVab positive (*N* = 54) during the whole experimental setup. Additionally, some individuals (c) seroconverted during the experiment (*N* = 54) while (d) few individuals converted from MORVab positive to negative (*N* = 5; see Mariën, Borremans, Gryseels, Vanden Broecke, et al., 2017). There were no significant differences in average weight among the four MORV classes (Table S3).

To disentangle the between‐ and within‐individual response of personality to density, we calculated the mean density for each individual (between‐individual level) and used within‐individual centering (deviation of one observation from the individuals' mean) for the within‐individual level (Dingemanse et al., 2012; van de Pol & Wright, 2009). Both were included as fixed effects together with all relevant two‐way interactions (Tables S1 and S2). We included the individuals' identity (ID), the enclosure where the individual was trapped in and the place where the individual originated from, since some individuals were originally trapped around SUA and released inside the enclosures before starting the experiment, as random effects in the model. By including ID as a random effect, we are able to calculate the repeatability of exploration and stress sensitivity (Nakagawa & Schielzeth, 2010). Statistically nonsignificant interactions and fixed effects were removed from the full model (Tables S1 and S2) using a backward stepwise procedure (*p* = .05 as the level to rejection) with the Kenward–Roger method (Luke, 2017) implemented in the R package lmerTest (version 3.0; Kuznetsova, Brockhoff, & Christensen, 2017). Significance of the random effects was tested using a likelihood ratio test (LRT) comparing the model with and without the random effect, a *p*‐value <.05 indicates that it explained a significant amount of the variance. However, we decided to keep all random factors in the model (even nonsignificant ones) since removing them could lead to pseudo‐replication and possibly an increased rate of type I error (Guenther, Brust, Dersen, & Trillmich, 2014). Individual variation in plasticity was estimated by creating a new LMM from the final model where we included the within‐individual centering of density as random slope (Dingemanse & Dochtermann, 2013), and significance was tested using the LRT.

#### 2.3.3 Effect of density and personality on MORVab

The effects of personality and density on MORVab presence were tested using a generalized linear mixed model (GLMM) with MORVab presence as a binary response variable (1 = positive, 0 = negative) and a binomial error structure (Crawley, 2012). Sex, reproductive age, and the two values of density (between‐ and within‐individual level) were included as fixed effects. We also examined the effects of personality on MORVab presence on both the between‐ and within‐individual level using the previously described method for both exploration and stress sensitivity and including them as fixed effects. Due to collinearity, we decided to not include any interactions into the model. Significance of the random effect was tested as previously described.

All continuous covariates were centered around their grand mean before analysis, and all statistical analyses were executed using R software 3.5.0 (R Core Team, 2013) with the R package lme4 (version 1.1; Bates, Mächler, Bolker, & Walker, 2015). Post hoc tests between categorical covariates were performed using the R package lsmeans (version 2.27; Lenth, 2016).

## 3 RESULTS

### 3.1 Behavioral analysis

The PCA reduced the number of behavioral variables to 2, explaining 70, 8% of the total variance (Table 1). The first component was positively correlated with activity and the two measurements of exploration and can therefore be seen as an activity‐exploration axis and will further be referred to as exploration. The second component was positively correlated with jumping during the behavioral test and negatively with self‐grooming (Table 1). Jumping during the behavioral test was clearly escaping behavior (Archer, 1973; Martin & Réale, 2008b). The interpretation of grooming is equivocal (Komorowska & Pisula, 2003), but during our observations, individuals that groomed a lot followed the cephalocaudal rule (grooming from the head to the tail), which occurs mostly in low stress environments (Smolinsky & Kalueff, 2011). We therefore argue that this behavioral axis represents the way in which the individual experiences stress and will call this behavioral axis “stress sensitivity.” Here, individuals that groom a lot are less stress sensitive than individuals that jumped more frequently.

**Table 1:** Correlation of each behavior observed during the hole‐board test with the components of the principal component

Component	PC1 (exploration)	PC2 (stress sensitivity)
Activity	**0.526**	−0.009
Head dip	**0.496**	−0.296
Sniffing	**0.565**	−0.324
Grooming	−0.331	**−0.523**
Jumping	0.221	**0.731**
Total variance (%)	47.1	23.7
Eigenvalue	2.355	1.185

Note

The two components were named, respectively: exploration and stress sensitivity.

Bold type indicates the behaviour that have a major contribution to a component.

### 3.2 Relationship between density and personality

**Figure 3:**
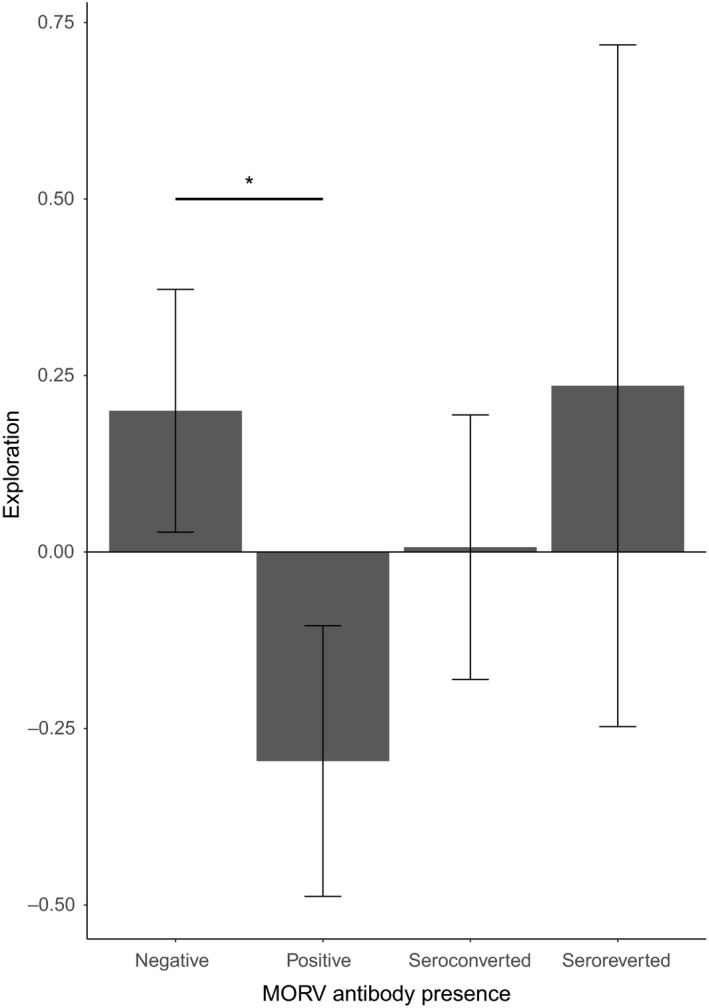
Differences in mean exploration behavior (± *SE*) among the four MORV classes. Individuals without antibodies against the MORV (*N* = 93) were significantly more explorative (*p* < .05) than individuals that were MORVab positive during the whole experiment (*N* = 54), which is indicated with * and the horizontal bar. Individuals that seroconverted (*N* = 54) or seroreverted (*N* = 5) did not differ significantly from the other MORV classes

The final LMM with exploration as a dependent variable revealed an interaction between test sequence and reproductive age (Figure S2) where adults habituated to the behavioral test (pairwise comparison: first‐subsequent recording: 0.795 ± 0.166, *t*_470_ = 4.778, *p* < .001) and became less active and explorative during subsequent recordings. Juveniles did not habituate (first‐subsequent recording: 0.167 ± 0.243, *t*_555_ = 0.690, *p* = .901) and were significantly more explorative than adults during subsequent recordings (Adult–Juvenile: −0.783 ± 0.186, *t*_630_ = −4.209, *p* < .001) but not during the first recording (adult–juvenile: −0.155 ± 0.240, *t*_642_ = −0.645, *p* = .917). Males were significantly more explorative (coefficient ± *SE* = 0.382 ± 0.144, *t*_200_ = 2.660, *p* = .008; Figure S2) than females. Additionally, we found significant differences among some of the MORVab classes (Figure 3), where MORVab positive (P) individuals were significantly less explorative than MORVab negative (N) individuals (N‐P: 0.496 ± 0.170, *t*_195_ = 2.920, *p* = .020). However, MORVab positive individuals did not differ from those that seroconverted (S) (P‐S: 0.303 ± 0.187, *t*_173_ = −1.621, *p* = .370) or seroreverted (L) (P‐L: −0.532 ± 0.479, *t*_228_ = −1.11, *p* = .683). Similarly, exploration behavior of MORVab negative individuals was not significantly different from those that seroconverted (N–S: 0.193 ± 0.165, *t*_180_ = 1.169, *p* = .647) or seroreverted (N‐L: −0.035 ± 0.474, *t*_229_ = −0.075, *p* = 1) and there were no differences between seroconverted and seroreverted individuals (S‐L: −0.229 ± 0.483, *t*_227_ = −0.473, *p* = .965).

The final LMM with stress sensitivity as a dependent variable also revealed a significant interaction between test sequence and reproductive age (Figure S1). Here, juveniles were more stress sensitive the first time they were measured (first‐subsequent: 0.536 ± 0.128, *t*_521_ = 4.202, *p* < .001) compared to subsequent recordings. Adults did not habituate (first‐subsequent: −0.213 ± 0.089, *t*_495_ = −2.382, *p* = .082) and did not differ significantly from juveniles when they were recorded again (adult–juvenile: 0.149 ± 0.129, *t*_658_ = 1.157, *p* = .654). However, adults were less stress sensitive than juveniles (adult–juvenile: −0.560 ± 0.133, *t*_638_ = −4.516, *p* < .001) when they were recorded for the first time.

**Figure 4:**
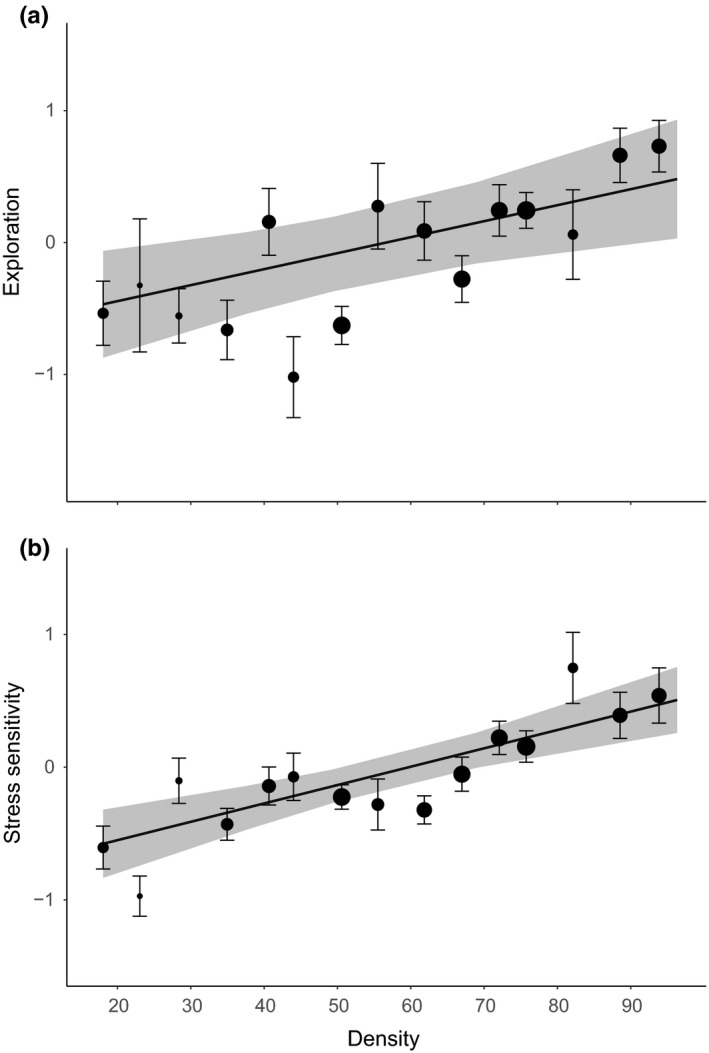
Linear correlation of both (a) stress sensitivity and (b) exploration with density at the between‐individual level. Predictions were made from the final LMM and the raw data of both stress sensitivity and exploration were superimposed as black circles with the diameter proportional to the number of sampling points where that mean density occurred, together with the standard error of that mean. The gray bar represents the standard error around the prediction

**Figure 5:**
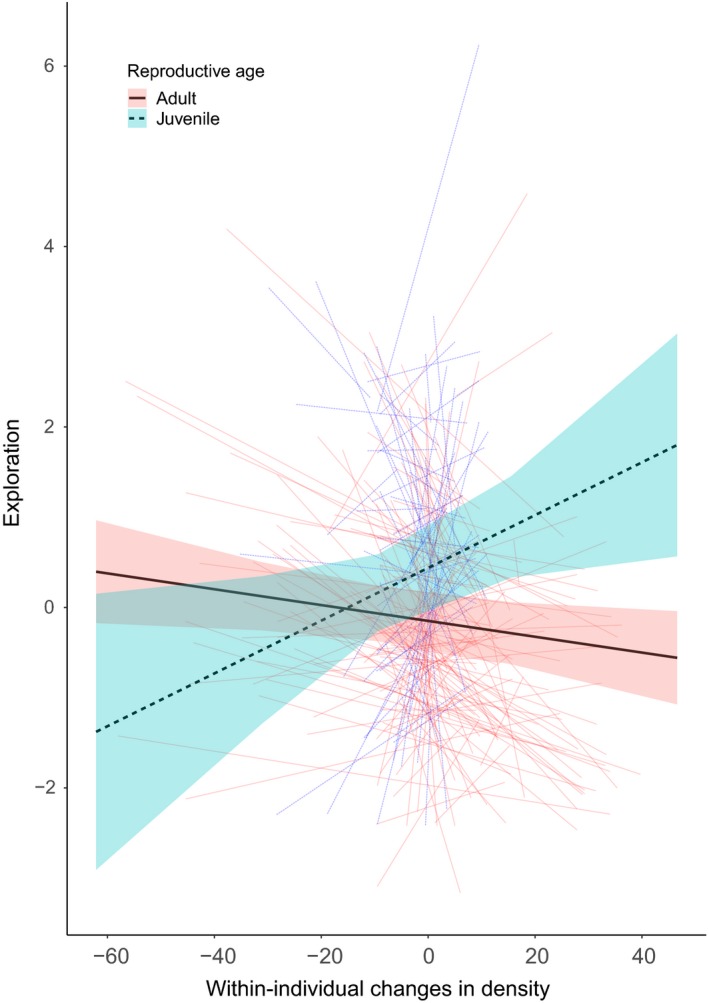
Correlation between exploration and density on the within‐individual level. The black lines are the predicted mean response of adults (solid line) and juveniles (dashed line) on the density changes they experienced during the experiment based on the final LMM. The colored bars around the mean response represent the standard error around the prediction. Each colored line represents an individuals' reaction on changing density, where the blue and red lines represent, respectively, juveniles and adults

Both exploration and stress sensitivity correlated significantly with density at the between‐individual level (Figure 4). When density increased we caught individuals that were on average more explorative (0.237 ± 0.082, *t*_86_ = 2.890, *p* = .039; Figure 4a) as well as individuals that were more stress sensitive (0.271 ± 0.057, *t*_221_ = 4.740, *p* = .005; Figure 4b). Only exploration correlated with density at the within‐individual level but differed between the two reproductive states (Figure 5). Adults became less explorative (−0.136 ± 0.064, *t*_343_ = −2.143, *p* = .033) when experiencing an increase in density and juveniles became more explorative (0.590 ± 0.204, *t*_605_ = 2.888, *p* = .004).

There were significant differences among individuals in both exploration and stress sensitivity (LRT: exploration: *χ* ^2^ = 32.216, *p* < .001; stress sensitivity: *χ* ^2^ = 108.340, *p* < .001). Exploration had a repeatability of *R* = 0.22 (95% CI 0.15–0.23) and stress sensitivity had a repeatability of *R* = 0.44 (95% CI 0.40–0.45). There were no differences among the three enclosures (exploration: *χ* ^2^ = 1.173, *p* = .279; stress sensitivity: *χ* ^2^ = 0, *p* = 1) nor did the area of origin explain a significant amount of the variation in either component (*χ* ^2^ = 0, *p* = 1). We found no indication for individual variation in plasticity (random slopes for individuals) with changing density for either components (exploration: *χ* ^2^ = 1.958, *p* = .376; stress sensitivity: *χ* ^2^ = 0.125, *p* = .939).

### 3.3 Effect of density and personality on MORVab

Our model revealed no significant differences in MORVab presence between the sexes (Table 2) nor between adults and juveniles (Table 2). Density did affect MORVab presence on both the between‐individual and the within‐individual level (Table 2), suggesting that seroprevalence increases with host density. Exploration negatively affected MORVab presence, independent of density on the between‐individual level, not on the within‐individual level (Table 2). Stress sensitivity had no effect on MORVab presence on both the between‐individual or the within‐individual level (Table 2). There were consistent differences between individuals in MORVab presence (*χ* ^2^ = 237.410, *p* < .001) but there were no differences among the enclosures (*χ* ^2^ = 1.404, *p* = .236) nor between areas of origin (*χ* ^2^ = 0.355, *p* = .552).

**Table 2:** Results from the generalized linear mixed model with MORV antibody status as a binomial dependent variable

	Estimate ± *SE*	*z*‐value	*p*‐value
Intercept	−1.883 ± 1.163	−1.619	.105
Sex (males)	0.556 ± 1.048	0.530	.596
Reproductive age (Juvenile)	−0.604 ± 0.662	−0.913	.361
Between‐individual level			
Exploration	−2.117 ± 0.699	−3.031	.002*
Stress sensitivity	−0.586 ± 0.542	−1.081	.280
Density	2.046 ± 0.944	2.168	.030*
Within‐individual level			
Exploration	−0.141 ± 0.171	−0.825	.410
Stress sensitivity	0.000 ± 0.163	0.003	.998
Density	1.528 ± 0.354	4.320	<.001*

Note

The method of van de Pol and Wright (2009) was used to disentangle the between‐ and within‐individual effects of exploration, stress sensitivity, and density on MORV antibody presence.

Significance is marked as follows:

* *p* < .05.

## 4 DISCUSSION

In this study, we found a positive correlation between MORVab presence and density at both the between‐ and the within‐individual level, resulting in more MORVab positive individuals when density increased. This suggests that transmission of MORV is density‐dependent and is in line with what has previously been suggested for this virus (Borremans et al., 2011, 2016; Borremans, Vossen, et al., 2015). Surprisingly, we found that personality, independently of density, correlated with MORV infection, where slower explorers were more likely to have antibodies against MORV compared to highly explorative individuals (Figure 3). Additionally, exploration was positively correlated with density on the between‐individual level (Figure 4a) which may suggest a negative, indirect effect of density on MORV infection.

### 4.1 Density and personality

We have shown here that *M. natalensis*, a short lived rodent species, expresses two separate personality traits inside the hole‐board test: exploration and stress sensitivity. Exploration has previously been described in *M. natalensis* (Vanden Broecke et al., 2018) and may provide the individual with information about the environment such as the availability of food. This is especially true during the breeding season when food is abundantly available (Leirs, 1995) and due to their lack of territoriality (Borremans et al., 2014) and generalist diet (Mulungu et al., 2011). It may also provide information about the availability of mates, specifically in males who use a scramble mating competition (Kennis et al., 2008), which could explain our results that males were significantly more explorative than females. Exploration correlated with density on both the between‐ and the within‐individual level. On the between‐individual level, we caught, on average, more explorative individuals when density increased, probably due to the addition of juveniles into the population (Leirs, Verhagen, & Verheyen, 1993; Leirs et al., 1994) which are more explorative than adults during subsequent recordings. Differences in exploration between reproductive stages have been found in several other taxa such as corvids (Miller, Bugnyar, Pölzl, & Schwab, 2015), brown rats (*Rattus norvegicus*; Ray & Hansen, 2005) as well in *M. natalensis* (Vanden Broecke et al., 2018), possibly because juveniles may have a greater need to be more explorative (Reader, 2015) which provides them with more information about the environment such as the availability of food resources. However, this need for information about resource availability might not be constant over time and change when individuals experience density variations.

Indeed, we found evidence for within‐individual plasticity in exploration behavior when density changed, but the direction in which they adjusted their exploration behavior differed between the two reproductive classes. Juveniles became more explorative with increasing density, while adults decreased their exploration behavior. These results suggest that both reproductive classes used different behavioural strategies to cope with changes in density, which could be explained by different future prospects. Juveniles mainly breed in the next breeding season after the one in which they were born (Leirs et al., 1993). Between these two breeding seasons is a prolonged period where food is scarce, since food depletion is one of the main sources of the population decrease in *M. natalensis* (Leirs et al., 1997). In order to increase their fitness and survival, juveniles will need to maximize their body weight before the growth stop at the end of the first breeding season (Kennis et al., 2008; Leirs, 1995; Leirs et al., 1990, 1993). Therefore, it is important that juveniles increase their exploration behavior with density if it helps them locating food resources. Adults, on the contrary, have a very low survival probability until the next breeding season (Leirs et al., 1993) where the benefits of increasing their exploration behavior do not outweigh the potential costs such as predator attraction (Jones & Godin, 2010; Rödel et al., 2015) and a higher metabolism (Careau & Garland, 2012; Careau, Thomas, Humphries, & Réale, 2008) and may potentially explain why adults do not increase their exploration with density. However, it is not clear if being more explorative increases survival. Nevertheless, the fact that there are differences in plasticity between reproductive classes suggest a potential cost of exploration and plasticity (Auld et al., 2010; DeWitt et al., 1998) and that exploration is more important than previously thought in *M. natalensis*, especially in juveniles.

The second personality trait, which we interpreted as stress sensitivity, was highly repeatable compared to other species (Bell, Hankison, & Laskowski, 2009; Bohn et al., 2017). This personality trait was strongly, positively, correlated with density on the between‐individual level where we caught more stress sensitive individuals with increasing density. One possible explanation is that this relationship is caused by changes in the social environment (Borremans et al., 2016). Martin and Réale (2008a, 2008b) found that Eastern chipmunks (*Tamias striatus*) whose home range overlapped with their neighbor, spend more time grooming during a hole‐board test than individuals without a neighbor, suggesting that social individuals groom more than others. If this would be the case in *M. natalensis*, we would expect to catch more individuals that groomed longer at higher density, since home range overlap and visitation rates increases with density (Borremans et al., 2014). However, larger home range overlaps do not necessarily imply more social contacts. Borremans et al. (2016) found that at low densities, *M. natalensis* increase their effort to maintain contacts with others, while at high density they avoid each other. It is therefore still possible that stress sensitivity is correlated with sociality, where less stress sensitive individuals might actively seek out other individuals at low density, while at higher densities, there are more stress sensitive individuals who actively avoid conspecifics.

### 4.2 Virus infection and personality

Surprisingly, we found that exploration correlated with MORV infection, independent of the effects of density, where MORVab positive individuals were significantly less explorative than MORVab negative individuals. These results contradict the hypothesis that fast explorers are more likely to get infected due to their risky lifestyle (Barber & Dingemanse, 2010), for which we provide four, nonmutually exclusive explanations. The first explanation is that our results are confounded with reproductive age, since adults are less explorative than juveniles (Vanden Broecke et al., 2018) but more likely to be MORVab positive (Borremans et al., 2011; Demby et al., 2001; Mariën, Borremans, Gryseels, Soropogui, et al., 2017; Mariën, Borremans, Gryseels, Vanden Broecke, et al., 2017). However, this explanation seems unlikely since we found no significant differences in weight between MORVab positive and negative individuals and reproductive age was not significant in the GLMM. A second explanation is that highly explorative individuals invest more in their immune system due to their increased encounter rate with parasites and pathogens (Barron, Gervasi, Pruitt, & Martin, 2015; Hulthen, Chapman, Nilsson, Hollander, & Bronmark, 2013; Kortet et al., 2010), or their immunity response is stronger because they are able to find more resources than less explorative individuals. This increased immunological investment provides protection against parasites and pathogens, including MORV, potentially increasing survival. Indeed, a long‐term study has shown that MORVab negative individuals have a higher survival probability than those with antibodies (Mariën et al., 2018). A third explanation is that sociability is negatively correlated with exploration in a behavioral syndrome, thereby reducing contact rates for highly explorative individuals (Sih, Bell, Johnson, & Ziemba, 2004). Indeed, this relationship has been proposed by Reale et al. (2010) but has rarely been studied and the results are equivocal (Haage, Bergvall, Maran, Kiik, & Angerbjörn, 2013; McCowan, Mainwaring, Prior, & Griffith, 2015; McEvoy, While, Sinn, Carver, & Wapstra, 2015; Thys et al., 2017). The fourth explanation is that MORV infection permanently alters the behavior of their host (Poulin, 2013), such as *Toxoplasma gondii* which reduces the overall activity of their rodent host (Piekarski, 1981). This could potentially explain why seroconverted individuals had intermediate levels of exploration. However, the GLMM showed that exploration and activity only correlated with MORVab presence at the between individual level, not at the within‐ individual level, which we should expect if infection alters behavior. To properly test this hypothesis, experimentally inoculating hosts should provide more insight.

Nonetheless, these results provide us with more insight into the virus dynamics of MORV within its natural host, which is currently understudied. MORV transmission has been found to be density‐dependent (Borremans et al., 2011, 2016; Borremans, Vossen, et al., 2015) and transmission between individuals increases after the breeding season. However, Goyens, Reijniers, Borremans, and Leirs (2013) observed a time lag between the positive correlation of MORV prevalence with density, for which we provide a potential additional explanation. Our results suggest that, when density increases, there are more fast exploring individuals in the population which are less likely to be infected with MORV. This may result in a growing proportion of susceptible individuals in the population in which the virus can invade after the breeding season (Goyens et al., 2013), which could result in the delayed density‐dependent transmission.

## 5 CONCLUSION

*Mastomys natalensis* experiences large changes in density over a very short temporal scale (Leirs et al., 1997, 1994; Sluydts et al., 2007) leading to changes in competitive and social environment (Borremans et al., 2016). This has strong effects on personality on both the between‐ and the within‐individual level. Our behavioral setup allowed us to separate these effects, compared to most studies who compared different populations experiencing different densities (Haigh et al., 2017; Korpela et al., 2011; Krebs, 1970), potentially ignoring spatial differences between them. We should note that we did not find behavioral differences in either exploration or stress sensitivity between the different enclosures even though they exhibited different densities through time. This suggests that our results are similar over the three populations, or replicates, and that the results are not biased due to unaccounted differences between the populations. Our results imply that density should be considered in further studies on wild populations.

While it has been suggested that personality might affect disease susceptibility and transmission (Barber & Dingemanse, 2010; Barron et al., 2015; Hawley, Etienne, Ezenwa, & Jolles, 2011), relatively little research has been done using viral models (Araujo, Kirschman, & Warne, 2016; Dizney & Dearing, 2013; Natoli et al., 2005). Our results show that there is indeed a link between personality and viral infection in our model system which changes with density and may provide us with a deeper understanding of the transmission mechanisms of this virus. Together, our results suggest that in order to better understand disease ecology and transmission, both personality and population density should be taken into account.

## AUTHOR CONTRIBUTIONS

BVB, EM, HL, and CS designed the study. BVB collected the data with support from JM, CS, LM, and AM. The labwork was performed by BVB and JM. BVB performed the data analysis with input from JM, EM, and HL. The first draft of the manuscript was written by BVB, and all authors contributed substantially to revisions. Finally, we thank the two anonymous referees for taking the time to review and improve this manuscript.

### OPEN RESEARCH BADGES

This article has earned an Open Data Badge for making publicly available the digitally‐shareable data necessary to reproduce the reported results. The data is available at https://doi.org/10.17605/OSF.IO/QJXNU.

## Data Availability

The data will be archived online in an appropriate public repository should the manuscript be accepted and the data DOI will be included at the end of the article.

## References

[ece35541-bib-0001] Altizer, S. , Dobson, A. , Hosseini, P. , Hudson, P. , Pascual, M. , & Rohani, P. (2006). Seasonality and the dynamics of infectious diseases. Ecology Letters, 9, 467–484. 10.1111/j.1461-0248.2005.00879.x 16623732

[ece35541-bib-0002] Anderson, R. M. , & May, R. M. (1978). Regulation and stability of host‐parasite population interactions: I. Regulatory processes. Journal of Animal Ecology, 47, 219.

[ece35541-bib-0003] Araujo, A. , Kirschman, L. , & Warne, R. W. (2016). Behavioural phenotypes predict disease susceptibility and infectiousness. Biology Letters, 12, 20160480 10.1098/rsbl.2016.0480 27555652 PMC5014041

[ece35541-bib-0004] Archer, J. (1973). Tests for emotionality in rats and mice: A review. Animal Behaviour, 21, 205–235. 10.1016/S0003-3472(73)80065-X 4578750

[ece35541-bib-0005] Auld, J. R. , Agrawal, A. A. , & Relyea, R. A. (2010). Re‐evaluating the costs and limits of adaptive phenotypic plasticity. Proceedings of the Royal Society B: Biological Sciences, 277, 503–511. 10.1098/rspb.2009.1355 PMC2842679 19846457

[ece35541-bib-0006] Barber, I. , & Dingemanse, N. J. (2010). Parasitism and the evolutionary ecology of animal personality. Philosophical Transactions of the Royal Society B: Biological Sciences, 365, 4077–4088. 10.1098/rstb.2010.0182 PMC2992744 21078659

[ece35541-bib-0007] Barron, D. , Gervasi, S. , Pruitt, J. , & Martin, L. (2015). Behavioral competence: How host behaviors can interact to influence parasite transmission risk. Current Opinion in Behavioral Sciences, 6, 35–40. 10.1016/j.cobeha.2015.08.002

[ece35541-bib-0008] Bates, D. , Mächler, M. , Bolker, B. , & Walker, S. (2015). Fitting linear mixed‐effects models using lme4. Journal of Statistical Software, 67, 1–48.

[ece35541-bib-0009] Bell, A. M. , Hankison, S. J. , & Laskowski, K. L. (2009). The repeatability of behaviour: A meta‐analysis. Animal Behaviour, 77, 771–783. 10.1016/j.anbehav.2008.12.022 24707058 PMC3972767

[ece35541-bib-0010] Blumstein, D. T. , & Daniel, J. C. (2007). Quantifying behavior the JWatcher way. Sunder‐land, MA: Sinauer Associates.

[ece35541-bib-0011] Bohn, S. J. , Webber, Q. M. R. , Florko, K. R. N. , Paslawski, K. R. , Peterson, A. M. , Piche, J. E. , … Willis, C. K. R. (2017). Personality predicts ectoparasite abundance in an asocial sciurid. Ethology, 123, 761–771. 10.1111/eth.12651

[ece35541-bib-0012] Borremans, B. , Hughes, N. K. , Reijniers, J. , Sluydts, V. , Katakweba, A. A. S. , Mulungu, L. S. , … Leirs, H. (2014). Happily together forever: Temporal variation in spatial patterns and complete lack of territoriality in a promiscuous rodent. Population Ecology, 56, 109–118. 10.1007/s10144-013-0393-2

[ece35541-bib-0013] Borremans, B. , Leirs, H. , Gryseels, S. , Günther, S. , Makundi, R. , & de Bellocq, J. G. (2011). Presence of Mopeia virus, an African arenavirus, related to biotope and individual rodent host characteristics: Implications for virus transmission. Vector‐Borne and Zoonotic Diseases, 11, 1125–1131. 10.1089/vbz.2010.0010 21142956

[ece35541-bib-0014] Borremans, B. , Reijniers, J. , Hughes, N. K. , Godfrey, S. S. , Gryseels, S. , Makundi, R. H. , & Leirs, H. (2016). Nonlinear scaling of foraging contacts with rodent population density. Oikos, 126, 792–800. 10.1111/oik.03623

[ece35541-bib-0015] Borremans, B. , Sluydts, V. , Makundi, R. H. , & Leirs, H. (2015). Evaluation of short‐, mid‐ and long‐term effects of toe clipping on a wild rodent. Wildlife Research, 42, 143–148. 10.1071/WR14109

[ece35541-bib-0016] Borremans, B. , Vossen, R. , Becker‐Ziaja, B. , Gryseels, S. , Hughes, N. , Van Gestel, M. , … Leirs, H. (2015). Shedding dynamics of Morogoro virus, an African arenavirus closely related to Lassa virus, in its natural reservoir host *Mastomys natalensis* . Scientific Reports, 5, 10445 10.1038/srep10445 26022445 PMC4448520

[ece35541-bib-0017] Boyer, N. , Réale, D. , Marmet, J. , Pisanu, B. , & Chapuis, J.‐L. (2010). Personality, space use and tick load in an introduced population of Siberian chipmunks *Tamias sibiricus* . Journal of Animal Ecology, 79, 538–547. 20202009 10.1111/j.1365-2656.2010.01659.x

[ece35541-bib-0018] Careau, V. , & Garland, T. (2012). Performance, personality, and energetics: Correlation, causation, and mechanism. Physiological and Biochemical Zoology, 85, 543–571. 10.1086/666970 23099454

[ece35541-bib-0019] Careau, V. , Thomas, D. , Humphries, M. , & Réale, D. (2008). Energy metabolism and animal personality. Oikos, 117, 641–653. 10.1111/j.0030-1299.2008.16513.x

[ece35541-bib-0020] Carere, C. , & Maestripieri, D. (2013). Animal personalities: Behavior, physiology and evolution. Chicago, IL: University of Chicago Press.

[ece35541-bib-0021] Clay, C. A. , Lehmer, E. M. , Previtali, A. , St Jeor, S. , & Dearing, M. D. (2009). Contact heterogeneity in deer mice: Implications for Sin Nombre virus transmission. Proceedings of the Royal Society B: Biological Sciences, 276, 1305–1312. 10.1098/rspb.2008.1693 PMC2660967 19129136

[ece35541-bib-0022] Crawley, M. J. (2012). The R book. Chichester, UK: John Wiley & Sons, Ltd.

[ece35541-bib-0023] Daszak, P. (2000). Emerging infectious diseases of wildlife– Threats to biodiversity and human health. Science, 287, 443–449. 10.1126/science.287.5452.443 10642539

[ece35541-bib-0024] Davis, S. , Begon, M. , De Bruyn, L. , Ageyev, V. S. , Klassovskiy, N. , Pole, S. B. , … Leirs, H. (2004). Predictive thresholds for plague in Kazakhstan. Science, 304, 736–738. 10.1126/science.1095854 15118163

[ece35541-bib-0025] Davis, S. , & Calvet, E. (2005). Fluctuating rodent populations and risk to humans from rodent‐borne zoonoses. Vector‐Borne and Zoonotic Diseases, 5, 305–314. 10.1089/vbz.2005.5.305 16417426

[ece35541-bib-0026] Demby, A. H. , Inapogui, A. , Kargbo, K. , Koninga, J. , Kourouma, K. , Kanu, J. , … Bausch, D. G. (2001). Lassa fever in Guinea: II. Distribution and prevalence of Lassa virus infection in small mammals. Vector‐Borne and Zoonotic Diseases, 1, 283–297. 10.1089/15303660160025912 12653128

[ece35541-bib-0027] DeWitt, T. J. , Sih, A. , & Wilson, D. S. (1998). Costs and limits of phenotypic plasticity. Trends in Ecology & Evolution, 13, 77–81. 10.1016/S0169-5347(97)01274-3 21238209

[ece35541-bib-0028] Dingemanse, N. J. , Bouwman, K. M. , van de Pol, M. , van Overveld, T. , Patrick, S. C. , Matthysen, E. , & Quinn, J. L. (2012). Variation in personality and behavioural plasticity across four populations of the great tit *Parus major* . Journal of Animal Ecology, 81, 116–126. 10.1111/j.1365-2656.2011.01877.x 21692798

[ece35541-bib-0029] Dingemanse, N. J. , & Dochtermann, N. A. (2013). Quantifying individual variation in behaviour: Mixed‐effect modelling approaches. Journal of Animal Ecology, 82, 39–54. 10.1111/1365-2656.12013 23171297

[ece35541-bib-0030] Dingemanse, N. J. , & Wolf, M. (2010). Recent models for adaptive personality differences: A review. Philosophical Transactions of the Royal Society B: Biological Sciences, 365, 3947–3958. 10.1098/rstb.2010.0221 PMC2992752 21078647

[ece35541-bib-0031] Dingemanse, N. J. , & Wolf, M. (2013). Between‐individual differences in behavioural plasticity within populations: Causes and consequences. Animal Behaviour, 85, 1031–1039. 10.1016/j.anbehav.2012.12.032

[ece35541-bib-0032] Dizney, L. , & Dearing, M. D. (2013). The role of behavioural heterogeneity on infection patterns: Implications for pathogen transmission. Animal Behaviour, 86, 911–916. 10.1016/j.anbehav.2013.08.003 PMC3850848 24319292

[ece35541-bib-0033] File, S. E. , & Wardill, A. G. (1975). Validity of head‐dipping as a measure of exploration in a modified hole‐board. Psychopharmacologia, 44, 53–59. 10.1007/BF00421184 1197580

[ece35541-bib-0034] Fischer, B. , van Doorn, G. S. , Dieckmann, U. , & Taborsky, B. (2014). The evolution of age‐dependent plasticity. The American Naturalist, 183, 108–125. 10.1086/674008 24334740

[ece35541-bib-0035] Frame, J. D. , Baldwin, J. M. , Gocke, D. J. , & Troup, J. M. (1970). Lassa fever, a new virus disease of man from West Africa. I. Clinical description and pathological findings. The American Journal of Tropical Medicine and Hygiene, 19, 670–676. 10.4269/ajtmh.1970.19.670 4246571

[ece35541-bib-0036] Goüy de Bellocq, J. , Borremans, B. , Katakweba, A. , Makundi, R. , Baird, S. J. E. , Becker‐Ziaja, B. , … Leirs, H. (2010). Sympatric occurrence of 3 Arenaviruses, Tanzania. Emerging Infectious Diseases, 16, 692–695. 10.3201/eid1604.091721 20350390 PMC3321973

[ece35541-bib-0037] Goyens, J. , Reijniers, J. , Borremans, B. , & Leirs, H. (2013). Density thresholds for Mopeia virus invasion and persistence in its host *Mastomys natalensis* . Journal of Theoretical Biology, 317, 55–61. 10.1016/j.jtbi.2012.09.039 23041432

[ece35541-bib-0038] Guenther, A. , Brust, V. , Dersen, M. , & Trillmich, F. (2014). Learning and personality types are related in cavies (*Cavia aperea*). Journal of Comparative Psychology, 128, 74–81. 10.1037/a0033678 24127657

[ece35541-bib-0039] Günther, S. , Hoofd, G. , Charrel, R. , Röser, C. , Becker‐Ziaja, B. , Lloyd, G. , … Leirs, H. (2009). Mopeia virus‐related arenavirus in natal multimammate mice, Morogoro, Tanzania. Emerging Infectious Diseases, 15, 2008–2012. 19961688 10.3201/eid1512.090864 PMC3044542

[ece35541-bib-0040] Haage, M. , Bergvall, U. A. , Maran, T. , Kiik, K. , & Angerbjörn, A. (2013). Situation and context impacts the expression of personality: The influence of breeding season and test context. Behavioural Processes, 100, 103–109. 10.1016/j.beproc.2013.08.009 23988476

[ece35541-bib-0041] Haigh, A. , O'Riordan, R. , & Butler, F. (2017). Variations in aggression and activity levels amongst squirrels inhabiting low and high density areas. Ecological Research, 32, 931–941. 10.1007/s11284-017-1506-8

[ece35541-bib-0042] Hawley, D. M. , Etienne, R. S. , Ezenwa, V. O. , & Jolles, A. E. (2011). Does animal behavior underlie covariation between hosts' exposure to infectious agents and susceptibility to infection? Implications for disease dynamics. Integrative and Comparative Biology, 51, 528–539. 10.1093/icb/icr062 21700577

[ece35541-bib-0043] Hulthen, K. , Chapman, B. B. , Nilsson, P. A. , Hollander, J. , & Bronmark, C. (2013). Express yourself: Bold individuals induce enhanced morphological defences. Proceedings of the Royal Society B: Biological Sciences, 281, 20132703. 10.1098/rspb.2013.2703 PMC3871320 24335987

[ece35541-bib-0044] Jones, K. A. , & Godin, J.‐G.‐J. (2010). Are fast explorers slow reactors? Linking personality type and anti‐predator behaviour. Proceedings of the Royal Society B: Biological Sciences, 277, 625–632. 10.1098/rspb.2009.1607 PMC2842688 19864291

[ece35541-bib-0045] Jones, K. E. , Patel, N. G. , Levy, M. A. , Storeygard, A. , Balk, D. , Gittleman, J. L. , & Daszak, P. (2008). Global trends in emerging infectious diseases. Nature, 451, 990–993. 10.1038/nature06536 18288193 PMC5960580

[ece35541-bib-0046] Kaiser, H. (1991). Coefficient alpha for a principal component and the Kaiser‐Guttman rule. Psychological Reports, 68, 855–858. 10.2466/pr0.1991.68.3.855

[ece35541-bib-0047] Karesh, W. B. , Dobson, A. , Lloyd‐Smith, J. O. , Lubroth, J. , Dixon, M. A. , Bennett, M. , … Heymann, D. L. (2012). Ecology of zoonoses: Natural and unnatural histories. Lancet, 380, 1936–1945. 10.1016/S0140-6736(12)61678-X 23200502 PMC7138068

[ece35541-bib-0048] Kennis, J. , Sluydts, V. , Leirs, H. , & van Hooft, W. F. P. (2008). Polyandry and polygyny in an African rodent pest species, *Mastomys natalensis* . Mammalia, 72, 150–160. 10.1515/MAMM.2008.025

[ece35541-bib-0049] Komorowska, J. , & Pisula, W. (2003). Does changing levels of stress affect the characteristics of grooming behavior in rats? International Journal of Comparative Psychology, 16, 237–246.

[ece35541-bib-0050] Korpela, K. , Sundell, J. , & Ylönen, H. (2011). Does personality in small rodents vary depending on population density? Oecologia, 165, 67–77. 10.1007/s00442-010-1810-2 20976607

[ece35541-bib-0051] Kortet, R. , Hedrick, A. V. , & Vainikka, A. (2010). Parasitism, predation and the evolution of animal personalities. Ecology Letters, 13, 1449–1458. 10.1111/j.1461-0248.2010.01536.x 21040352

[ece35541-bib-0052] Krebs, C. J. (1970). Microtus population biology: Behavioral changes associated with the population cycle in *M. ochrogaster* and *M. pennsylvanicus* . Ecology, 51, 34–52. 10.2307/1933598

[ece35541-bib-0053] Kuznetsova, A. , Brockhoff, P. B. , & Christensen, R. H. B. (2017). lmerTest package: Tests in linear mixed effects models. Journal of Statistical Software, 82, 26.

[ece35541-bib-0054] Le Galliard, J.‐F. , Paquet, M. , & Mugabo, M. (2015). An experimental test of density‐dependent selection on temperament traits of activity, boldness and sociability. Journal of Evolutionary Biology, 28, 1144–1155. 10.1111/jeb.12641 25865798

[ece35541-bib-0055] Leirs, H. (1994). Population ecology of *Mastomys natalensis* (Smith, 1834). Implications for rodent control in Africa. Brussels, Belgium: Belgian Administration for Development Cooperation.

[ece35541-bib-0056] Leirs, H. (1995). Population ecology of *Mastomys natalensis* (Smith, 1834). Implications for rodent control in Africa. A report from the Tanzania‐Belgium Joint Rodent Research Project (1986–1989). Publ. Agric.

[ece35541-bib-0057] Leirs, H. , Stenseth, N. C. , Nichols, J. D. , Hines, J. E. , Verhagen, R. , & Verheyen, W. (1997). Stochastic seasonality and nonlinear density‐dependent factors regulate population size in an African rodent. Nature, 389, 176–180. 10.1038/38271 9296494

[ece35541-bib-0058] Leirs, H. , Stuyck, J. , Verhagen, R. , & Verheyen, W. (1990). Seasonal variation in growth of *Mastomys natalensis* (Rodentia: Muridae) in Morogoro, Tanzania. African Journal of Ecology, 28, 298–306. 10.1111/j.1365-2028.1990.tb01164.x

[ece35541-bib-0059] Leirs, H. , Verhagen, R. , & Verheyen, W. (1993). Productivity of different generations in a population of *Mastomys natalensis* rats in Tanzania. Oikos, 68, 53–60. 10.2307/3545308

[ece35541-bib-0060] Leirs, H. , Verhagen, R. , & Verheyen, W. (1994). The basis of reproductive seasonality in Mastomys rats (Rodentia: Muridae) in Tanzania. Journal of Tropical Ecology, 10, 55–66.

[ece35541-bib-0061] Lenth, R. V. (2016). Least‐squares means: The R package lsmeans. Journal of Statistical Software, 69, 1–33.

[ece35541-bib-0062] Luke, S. G. (2017). Evaluating significance in linear mixed‐effects models in R. Behavior Research Methods, 49, 1494–1502. 10.3758/s13428-016-0809-y 27620283

[ece35541-bib-0063] Mariën, J. (2019). Transmission ecology of old world arenaviruses in natural populations of their reservoir hosts. Universiteit Antwerpen, Faculteit Wetenschappen, Departement Biologie, Antwerpen, Belgium.

[ece35541-bib-0064] Mariën, J. , Borremans, B. , Gryseels, S. , Soropogui, B. , De Bruyn, L. , Bongo, G. N. , … Fichet‐Calvet, E. (2017). No measurable adverse effects of Lassa, Morogoro and Gairo arenaviruses on their rodent reservoir host in natural conditions. Parasites & Vectors, 10, 210 10.1186/s13071-017-2146-0 28449693 PMC5408478

[ece35541-bib-0065] Mariën, J. , Borremans, B. , Gryseels, S. , Vanden Broecke, B. , Becker‐Ziaja, B. , Makundi, R. , … Leirs, H. (2017). Arenavirus dynamics in experimentally and naturally infected rodents. EcoHealth, 14, 463–473. 10.1007/s10393-017-1256-7 28616660

[ece35541-bib-0066] Mariën, J. , Sluydts, V. , Borremans, B. , Gryseels, S. , Vanden Broecke, B. , Sabuni, C. A. , … Leirs, H. (2018). Arenavirus infection correlates with lower survival of its natural rodent host in a long‐term capture‐mark‐recapture study. Parasites & Vectors, 11, 90 10.1186/s13071-018-2674-2 29422075 PMC5806307

[ece35541-bib-0067] Martin, J. G. A. , & Réale, D. (2008a). Animal temperament and human disturbance: Implications for the response of wildlife to tourism. Behavioural Processes, 77, 66–72. 10.1016/j.beproc.2007.06.004 17683881

[ece35541-bib-0068] Martin, J. G. A. , & Réale, D. (2008b). Temperament, risk assessment and habituation to novelty in eastern chipmunks, *Tamias striatus* . Behavioural Processes, 75, 309–318. 10.1016/j.anbehav.2007.05.026

[ece35541-bib-0069] McCowan, L. S. C. , Mainwaring, M. C. , Prior, N. H. , & Griffith, S. C. (2015). Personality in the wild zebra finch: Exploration, sociality, and reproduction. Behavioral Ecology, 26, 735–746. 10.1093/beheco/aru239

[ece35541-bib-0070] McEvoy, J. , While, G. M. , Sinn, D. L. , Carver, S. , & Wapstra, E. (2015). Behavioural syndromes and structural and temporal consistency of behavioural traits in a social lizard. Journal of Zoology, 296, 58–66. 10.1111/jzo.12217

[ece35541-bib-0071] Meijering, E. , Dzyubachyk, O. , & Smal, I. (2012). Methods for cell and particle tracking In Michael Conn P. (Ed.), Methods enzymol (pp. 183–200). Cambridge, MA: Academic Press. 10.1016/B978-0-12-391857-4.00009-4 22264535

[ece35541-bib-0072] Miller, R. , Bugnyar, T. , Pölzl, K. , & Schwab, C. (2015). Differences in exploration behaviour in common ravens and carrion crows during development and across social context. Behavioral Ecology and Sociobiology, 69, 1209–1220. 10.1007/s00265-015-1935-8 26097282 PMC4464646

[ece35541-bib-0073] Mohr, K. , Vibe‐Petersen, S. , Lau Jeppesen, L. , Bildsoe, M. , & Leirs, H. (2003). Foraging of multimammate mice, *Mastomys natalensis*, under different predation pressure: Cover, patch‐dependent decisions and density‐dependent GUDs. Oikos, 100, 459–468. 10.1034/j.1600-0706.2003.11763.x

[ece35541-bib-0074] Mulungu, L. S. , Mahlaba, T. A. , Massawe, A. W. , Kennis, J. , Crauwels, D. , Eiseb, S. , … Belmain, S. R. (2011). Dietary differences of the multimammate mouse, *Mastomys natalensis* (Smith, 1834), across different habitats and seasons in Tanzania and Swaziland. Wildlife Research, 38, 640–646. 10.1071/WR11028

[ece35541-bib-0075] Nakagawa, S. , & Schielzeth, H. (2010). Repeatability for Gaussian and non‐Gaussian data: A practical guide for biologists. Biological Reviews, 85, 935–956. 10.1111/j.1469-185X.2010.00141.x 20569253

[ece35541-bib-0076] Natoli, E. , Say, L. , Cafazzo, S. , Bonanni, R. , Schmid, M. , & Pontier, D. (2005). Bold attitude makes male urban feral domestic cats more vulnerable to Feline Immunodeficiency Virus. Neuroscience & Biobehavioral Reviews, 29, 151–157. 10.1016/j.neubiorev.2004.06.011 15652262

[ece35541-bib-0077] Nicolaus, M. , Tinbergen, J. M. , Ubels, R. , Both, C. , & Dingemanse, N. J. (2016). Density fluctuations represent a key process maintaining personality variation in a wild passerine bird. Ecology Letters, 19, 478–486. 10.1111/ele.12584 26929092

[ece35541-bib-0078] Patterson, L. D. , & Schulte‐Hostedde, A. I. (2011). Behavioural correlates of parasitism and reproductive success in male eastern chipmunks, *Tamias striatus* . Animal Behaviour, 81, 1129–1137. 10.1016/j.anbehav.2011.02.016

[ece35541-bib-0079] Peres‐Neto, P. R. , Jackson, D. A. , & Somers, K. M. (2005). How many principal components? Stopping rules for determining the number of non‐trivial axes revisited. Computational Statistics & Data Analysis, 49, 974–997. 10.1016/j.csda.2004.06.015

[ece35541-bib-0080] Piekarski, G. (1981). Behavioral alterations caused by parasitic infection in case of latent toxoplasma infection. Zentralblatt Für Bakteriologie, Mikrobiologie Und Hygiene. 1. Abt. Originale A, Medizinische Mikrobiologie, Infektionskrankheiten Und Parasitologie, 250, 403–406. 7197863

[ece35541-bib-0081] Poulin, R. (2013). Parasite manipulation of host personality and behavioural syndromes. Journal of Experimental Biology, 216, 18–26. 10.1242/jeb.073353 23225863

[ece35541-bib-0082] R Core Team (2013). R: A language and environment for statistical computing. Vienna, Austria: R Foundation for Statistical Computing.

[ece35541-bib-0083] Ray, J. , & Hansen, S. (2005). Temperamental development in the rat: The first year. Developmental Psychobiology, 47, 136–144. 10.1002/dev.20080 16136549

[ece35541-bib-0084] Reader, S. M. (2015). Causes of individual differences in animal exploration and search. Topics in Cognitive Science, 7, 451–468. 10.1111/tops.12148 25982255

[ece35541-bib-0085] Reale, D. , Garant, D. , Humphries, M. M. , Bergeron, P. , Careau, V. , & Montiglio, P.‐O. (2010). Personality and the emergence of the pace‐of‐life syndrome concept at the population level. Philosophical Transactions of the Royal Society B: Biological Sciences, 365, 4051–4063. 10.1098/rstb.2010.0208 PMC2992747 21078657

[ece35541-bib-0086] Réale, D. , Reader, S. M. , Sol, D. , McDougall, P. T. , & Dingemanse, N. J. (2007). Integrating animal temperament within ecology and evolution. Biological Reviews, 82, 291–318. 10.1111/j.1469-185X.2007.00010.x 17437562

[ece35541-bib-0087] Rödel, H. G. , Zapka, M. , Talke, S. , Kornatz, T. , Bruchner, B. , & Hedler, C. (2015). Survival costs of fast exploration during juvenile life in a small mammal. Behavioral Ecology and Sociobiology, 69, 205–217. 10.1007/s00265-014-1833-5

[ece35541-bib-0088] Schneider, C. A. , Rasband, W. S. , & Eliceiri, K. W. (2012). NIH Image to ImageJ: 25 years of image analysis. Behavioral Ecology and Sociobiology, 9, 671–675. 10.1038/nmeth.2089 PMC5554542 22930834

[ece35541-bib-0089] Sih, A. , Bell, A. M. , Johnson, J. C. , & Ziemba, R. E. (2004). Behavioral syndromes: An integrative overview. The Quarterly Review of Biology, 79, 241–277. 10.1086/422893 15529965

[ece35541-bib-0090] Sluydts, V. , Crespin, L. , Davis, S. , Lima, M. , & Leirs, H. (2007). Survival and maturation rates of the African rodent, *Mastomys natalensis*: Density‐dependence and rainfall. Integrative Zoology, 2, 220–232. 21396039 10.1111/j.1749-4877.2007.00065.x

[ece35541-bib-0091] Sluydts, V. , Davis, S. , Mercelis, S. , & Leirs, H. (2009). Comparison of multimammate mouse (*Mastomys natalensis*) demography in monoculture and mosaic agricultural habitat: Implications for pest management. Crop Protection, 28, 647–654. 10.1016/j.cropro.2009.03.018

[ece35541-bib-0092] Smolinsky, A. N. , & Kalueff, A. V. (2011). Analysis of grooming behavior and its utility in studying animal stress, anxiety, and depression In Gould T. D. (Ed.), Mood and anxiety related phenotypes in mice, Neuromethods (pp. 21–36). Totowa, NJ: Humana Press.

[ece35541-bib-0093] Thys, B. , Eens, M. , Aerts, S. , Delory, A. , Iserbyt, A. , & Pinxten, R. (2017). Exploration and sociability in a highly gregarious bird are repeatable across seasons and in the long term but are unrelated. Animal Behavior, 123, 339–348.

[ece35541-bib-0094] van de Pol, M. , & Wright, J. (2009). A simple method for distinguishing within‐ versus between‐subject effects using mixed models. Animal Behaviour, 77, 753–758. 10.1016/j.anbehav.2008.11.006

[ece35541-bib-0095] Vanden Broecke, B. , Borremans, B. , Mariën, J. , Makundi, R. H. , Massawe, A. W. , Leirs, H. , & Hughes, N. K. (2018). Does exploratory behavior or activity in a wild mouse explain susceptibility to virus infection? Current Zoology, 64, 585–592. 30323837 10.1093/cz/zox053 PMC6178786

[ece35541-bib-0096] VanderWaal, K. L. , & Ezenwa, V. O. (2016). Heterogeneity in pathogen transmission: Mechanisms and methodology. Functional Ecology, 30, 1606–1622. 10.1111/1365-2435.12645

[ece35541-bib-0097] Wolf, M. , van Doorn, G. S. , Leimar, O. , & Weissing, F. J. (2007). Life‐history trade‐offs favour the evolution of animal personalities. Nature, 447, 581–584. 10.1038/nature05835 17538618

[ece35541-bib-0098] Woolhouse, M. E. J. , Dye, C. , Etard, J.‐F. , Smith, T. , Charlwood, J. D. , Garnett, G. P. , … Anderson, R. M. (1997). Heterogeneities in the transmission of infectious agents: Implications for the design of control programs. Proceedings of the National Academy of Sciences of the United States of America, 94, 338–342. 10.1073/pnas.94.1.338 8990210 PMC19338

[ece35541-bib-0099] Ziwa, M. H. , Matee, M. I. , Kilonzo, B. S. , & Hang'ombe, B. M. (2013). Evidence of Yersinia pestis DNA in rodents in plague outbreak foci in Mbulu and Karatu Districts, northern Tanzania. Tanzania Journal of Health Research, 15, 1–8. 10.4314/thrb.v15i3.1 26591703

