# Supplementary material for: Relationship between population density and viral infection: A role for personality?
Source: Ecol Evol. 2019 Aug 18;9(18):10213–24. doi: 10.1002/ece3.5541 (PMC6787790; doi:10.1002/ece3.5541)

**Supporting information**

Table 1: Results from the stepwise reduction of the full linear mixed model (LMM) of exploration (the first principal component).

|  | Exploration (PC1) | | |
| --- | --- | --- | --- |
| Independent variable | *df* | *F* | *P* |
| Test sequence x sex | 1, 515 | 0.006 | 0.939 |
| MORVab x sex | 3, 194 | 0.140 | 0.936 |
| Density (within-individual) x sex | 1, 484 | 0.012 | 0.912 |
| Density (between-individual) x sex | 1, 232 | 0.032 | 0.859 |
| Test sequence x MORVab | 3, 513 | 0.366 | 0.778 |
| Reproductive age x sex | 1, 559 | 0.436 | 0.509 |
| Reproductive age x density (between-individual) | 1, 262 | 0.801 | 0.372 |
| MORVab x density (between-individual) | 3, 219 | 1.058 | 0.368 |
| Reproductive age x MORVab | 3, 541 | 1.674 | 0.171 |
| MORVab x density (within-individual) | 3, 459 | 1.651 | 0.177 |
| MORVab ^a^ | 3, 199 | 2.869 | 0.038 * |
| Sex ^a^ | 1, 203 | 6.957 | 0.009 * |
| Density (between-individual) ^a^ | 1, 10 | 5.535 | 0.040 * |
| Reproductive age x density (within-individual) ^a^ | 1, 605 | 8.235 | 0.004 * |
| Reproductive age x test sequence ^a^ | 1, 555 | 4.443 | 0.035 * |

Notes: significance is marked as follows: **P*<0.05

^a^ Final model.

Table 2: Results from the stepwise reduction of the full linear mixed model (LMM) of Stress sensitivity (the second principal component).

|  | Stress sensitivity (PC2) | | |
| --- | --- | --- | --- |
| Independent variable | df | F | P |
| MORVab x density (within-individual) | 3, 454 | 0.167 | 0.919 |
| Reproductive age x density (between-individual) | 1, 191 | 0.043 | 0.835 |
| Density (between-individual) x sex | 1, 216 | 0.127 | 0.722 |
| Density (within-individual) x sex | 1, 465 | 0.193 | 0.661 |
| MORVab x sex | 3, 201 | 0.616 | 0.606 |
| Reproductive age x density (within-individual) | 1, 556 | 1.041 | 0.308 |
| Density (within-individual) | 1, 228 | 0.609 | 0.436 |
| MORVab x density (between-individual) | 3, 205 | 1.337 | 0.263 |
| Reproductive age x sex | 1, 620 | 1.665 | 0.197 |
| Test sequence x sex | 1, 471 | 0.804 | 0.370 |
| Sex | 1, 203 | 0.141 | 0.708 |
| Reproductive age x MORVab | 3, 625 | 1.906 | 0.127 |
| Test sequence x MORVab | 3, 455 | 1.465 | 0.223 |
| MORVab | 3, 201 | 1.012 | 0.388 |
| Density (between-individual) ^a^ | 1, 10 | 13.148 | 0.005 * |
| Reproductive age x test sequence ^a^ | 1, 249 | 20.813 | <0.001 * |

Notes: significance is marked as follows: **P*<0.05

^a^ Final model.

Table 3: Results from the linear mixed model with weight as dependent variable, to determine differences in weight among the four MORV antibody classes.

|  | **Estimate ± SE** | **t-value** | **P-value** |
| --- | --- | --- | --- |
| Intercept | 40.048 ± 4.460 | 8.979 | < 0.001 * |
| Sex (males) | -1.775 ± 1.388 | -1.279 | 0.203 |
| Reproductive age (Juvenile) | -22.971 ± 1.013 | -22.686 | < 0.001 * |
| MORVab |  |  |  |
| Negative | 3.482 ± 4.478 | 0.778 | 0.438 |
| Positive | 4.477 ± 4.553 | 0.983 | 0.327 |
| Seroconverted | 7.151 ± 4.558 | 1.569 | 0.118 |

Notes: significance is marked as follows: **P*<0.05

Table 4: Dates when we conducted the capture-mark-recapture trapping in each enclosure. For each session we also noted the abundance (minimal animals alive).

| **Session** | **Trap Dates** | **Abundance** |
| --- | --- | --- |
| Enclosure A | | |
| 1 | 15-17 Aug '17 | 39 |
| 2 | 29-31 Aug '17 | 38 |
| 3 | 12-14 Sep '17 | 34 |
| 4 | 26-28 Sep '17 | 39 |
| 5 | 11-13 Oct '17 | 58 |
| Enclosure B | | |
| 1 | 26-30 Jul '17 | 23 |
| 2 | 11-13 Jul '17 | 19 |
| 3 | 25-27 Jul '17 | 25 |
| 4 | 08-10 Aug '17 | 44 |
| 5 | 22-24 Aug '17 | 57 |
| 6 | 05-07 Sep '17 | 67 |
| 7 | 19-21 Sep '17 | 71 |
| 8 | 03-05 Oct '17 | 60 |
| 9 | 17-19 Oct '17 | 78 |
| Enclosure C | | |
| 1 | 25-27 Jul '17 | 30 |
| 2 | 08-10 Aug '17 | 36 |
| 3 | 22-24 Aug '17 | 40 |
| 4 | 05-07 Sep '17 | 72 |
| 5 | 19-21 Sep '17 | 85 |
| 6 | 03-05 Oct '17 | 86 |
| 7 | 17-19 Oct '17 | 93 |

Figure legends

Figure 1

Mean scores (± SE) of (A) exploration and (B) stress sensitivity of adults and juveniles when they were recorded for the first time compared to subsequent recordings.

Figure 2

Differences in mean exploration behaviour (± SE) between males and females.

Figure 1


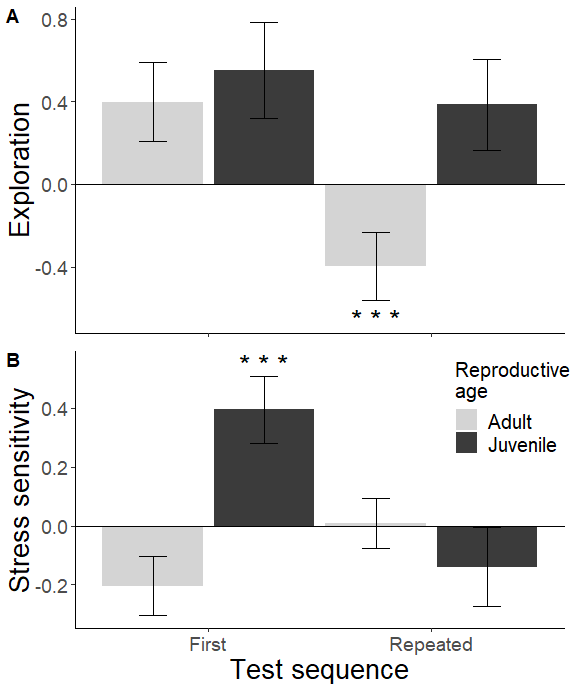


Figure 2


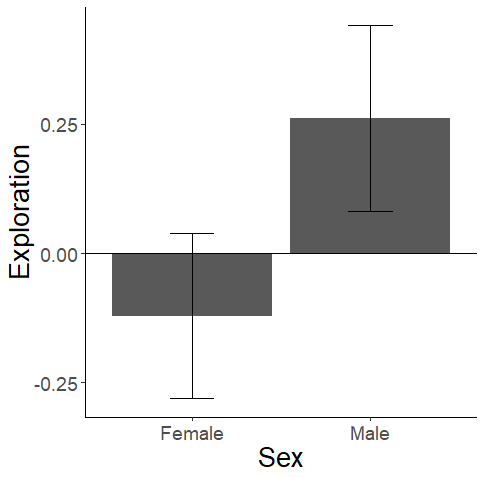

Supplement: Supplementary file 1 [file ECE3-9-10213-s001.docx]
